# Facial expressions of pain: the role of the serotonergic system

**DOI:** 10.1007/s00213-023-06455-y

**Published:** 2023-09-07

**Authors:** Miriam Kunz, Karl-Jürgen Bär, Anna J. Karmann, Gerd Wagner, Stefan Lautenbacher

**Affiliations:** 1 Department of Medical Psychology and Sociology, Medical Faculty, University of Augsburg, Augsburg, Germany; 2 Bamberger Living Lab Dementia (BamLiD), Otto-Friedrich University Bamberg, Bamberg, Germany; 3 Department of Psychosomatic Medicine and Psychotherapy, Jena University Hospital, Jena, Germany; 4 Department of Psychiatry and Psychotherapy, Jena University Hospital, Jena, Germany

**Keywords:** Facial responses, Facial expression of pain, 5-HT, Acute tryptophan depletion, Pain thresholds

## Abstract

**Rationale:**

Although interest in the neurobiology of facial communication of pain has increased over the last decades, little is known about which neurotransmitter systems might be involved in regulating facial expressions of pain.

**Objectives:**

We aim to investigate whether the serotonergic system (5-HT), which has been implicated in various aspects of pain processing as well as in behavioral response inhibition, might play a role in facial expressions of pain. Using acute tryptophan depletion (ATD) to manipulate 5-HT function, we examined its effects on facial and subjective pain responses.

**Methods:**

In a double-blind, placebo-controlled within-subject design, 27 participants received either an ATD or a control drink in two separate sessions. Approximately 5-h post-oral consumption, we assessed pain thresholds (heat, pressure) as well as facial and subjective responses to phasic heat pain. Moreover, situational pain catastrophizing and mood were assessed as affective state indicators.

**Results:**

ATD neither influenced pain thresholds nor self-report ratings, nor catastrophizing or mood. Only facial responses were significantly affected by ATD. ATD led to a decrease in pain-indicative as well as in pain-non-indicative facial responses to painful heat, compared to the control condition.

**Conclusions:**

Decrease in brain 5-HT synthesis via ATD significantly reduced facial responses to phasic heat pain; possibly due to (i) diminished disposition to display social behavior or due to (ii) decreased facilitation of excitatory inputs to the facial motor neuron.

## Introduction

Facial expressions of pain play an important part in social interactions as well as in clinical settings (Craig et al. 2011; Kunz et al. 2007). Over the last decades, research interest in this behavioral indicator of pain and its underlying biopsychosocial mechanisms has substantially increased (Hadjistavropoulos et al. 2011). However, the neurobiological basis is still largely unknown with the exception of some findings on the functional neuroanatomy. It has been shown that next to primary motor areas and pain-related areas (primary somatosensory cortex, insula), facial expressions of pain are also closely linked to activation in prefrontal areas of the brain which seem to be involved in inhibiting or gating the facial expression (Karmann et al. 2016; Kunz et al. 2011; 2020).

In contrast, there is a substantial lack of knowledge with regard to neurotransmitter systems being involved in facial expressions of pain. An interesting candidate in this context might be the serotonergic system. First of all, serotonin (5-HT) plays an important role in nociceptive processing; among others, via descending inhibitory serotonergic projections from the nucleus raphe magnus to the spinal cord (Basbaum & Fields 1984; Feng et al. 2012). As animal studies have shown, these descending serotonergic projections can both inhibit as well as facilitate nociception, depending on the site of action and on the receptor subtype (Martins 2019). Given this complexity of action, it is not surprising that the role of 5-HT in experimental and clinical pain could still not be finally clarified in human studies, although there is some indication that low central levels of 5-HT might be associated with increased clinical pain complaints (Goesling et al. 2013; Cooper et al. 2017, Nagata et al. 2007). A well-established method to experimentally investigate the role of 5-HT in humans is acute tryptophan depletion (ATD; Crockett et al. 2012; Young 2013); with tryptophan (Trp) being the direct precursor of 5-HT. ATD leads to a reduction in central 5-HT synthesis between 4 and 7 h after oral administration of an amino acid mixture omitting tryptophan (Carpenter et al. 1998). Using ATD, it was shown that the acute decrease in 5-HT synthesis leads to enhanced experimental pain responses, as indicated by reduced heat pain thresholds and tolerance (Martin et al. 2017) and reduced cold pain tolerance, but not threshold (Trotter et al. 2022). Thus, although contradictory findings have also been found (Abbott et al. 1992); ATD and hereby low levels of central 5-HT mainly seem to be associated with increased pain sensitivity.

Second, besides its role in pain processing, 5-HT also seems to affect behavioral inhibition and impulse control (Cools et al. 2008). Using ATD, it was shown that reduced central 5-HT was associated with disinhibition of goal-directed behavioral responses to reward (Walderhaug et al. 2002; 2008; Crockett et al. 2009) which seems to co-occur with activity changes in the prefrontal cortex (Aquili 2020). Although facial expressions of pain are more reflex-like responses, they are also under inhibitory control, with prefrontal structures gating the degree of facial inhibition (Karmann et al. 2015, 2016; Kunz et al. 2011; 2020) due to learned social display rules (Larochette et al. 2006). According to these considerations, it is possible that ATD might also lead to a disinhibition of facial expressions of pain.

Taken together, 5-HT has the potential to modulate facial expressions of pain at least via two mechanisms, namely via changes in nociceptive processing as well as via changes in behavioral inhibition. The aim of the present study was to use ATD to investigate the role of 5-HT in facial expressions of pain in response to experimental pain. We hypothesized that ATD and thus, low levels of central 5-HT, will result in increased facial expressions of pain because of both a lowering of nociceptive inhibition and a reduction of behavioral inhibition. For control, ATD related changes in pain ratings and in affective states were also assessed.

## Materials and methods

### Participants

Thirty female participants were recruited by bulletins put up throughout the campus of the University of Jena. Only women were recruited given previous evidence showing elevated behavioral as well as biochemical ATD responses in females compared to males (Ellenbogen et al. 1996; Nishizawa et al. 1997). None of the participants had present or past history of psychiatric or neurological disorders (assessed using the German version of the Mini-International Neuropsychiatric Interview (MINI) (Sheehan et al. 1998)) and none had acute or chronic pain. All participants were right-handed. Three participants were excluded due to nausea occurring after consumption of the used amino acid mixture (ATD: *N* = 2; control: *N* = 1). Thus, the final sample consisted of 27 participants (age: mean = 24.1 ± 4.4 years). Informed written consent was obtained in accordance with the protocols approved by the Ethics Committee of the University of Jena and all participants received monetary compensation (50€) for their participation.

### Design

Acute tryptophan depletion (ATD) was applied in a double-blind, placebo-controlled crossover design; thus, each participant was tested under the ATD (Trp −) and the control condition (Trp +). The order of both conditions was randomized and counterbalanced across participants. The two conditions were separated by a 7-day period to rule out potential carry-over effects.

### Procedure

Participants were required to fast, starting from 7 p.m. the day prior to the experiment, with the exception of water consumption. Upon arrival, a first blood sample was taken (10 ml) to assess baseline tryptophan levels. Participants were then administered the amino acid beverage (ADT or control). Following the oral consumption, participants were asked to wait in a designated waiting area for 4 h, given that the maximum depletion effect takes at least 4 h to develop (Williams et al. 1999). During the waiting time, a study nurse regularly checked on the participants. At the end of the waiting time, participants completed the Self-Assessment Manikin (SAM, Bradley & Lang 1994) for mood assessment (valence ratings). Moreover, a second blood sampling was conducted to again assess tryptophan levels. Directly afterward, participants took part in a 30–45 min MRI (magnetic resonance imaging) brain scan (these findings have been reported previously; Bär et al. 2020), followed by the pain testing block.

### Acute tryptophan depletion

ATD inhibits serotonin synthesis by lowering the availability of the serotonin precursor, tryptophan. This is achieved by participants consuming a tryptophan-free amino acid drink, which results in a substantial decline in plasma tryptophan (Trp) levels, with peak depletion occurring approximately 4–7 h later (Williams et al. 1999, Carpenter et al. 1998). For this study, we used an ATD mixture and a balanced control mixture which contained all amino acids. The compositions of the drinks have been described in detail before (Bär et al. 2020). In short, we used a fixed collagen-based amino acid mixture following established methods by Evers et al. (2005). The powder was purchased from Abtei OP Pharma GmbH. The control mixture (Trp +) differed from the composition of the ATD mixture only in that the mixture was supplemented by l-tryptophan of 10.3 g (Young et al. 1985). The powdered amino acids were mixed with 300 ml of water a few minutes prior to oral administration.

Venous blood samples (taken pre- and 4-h post-oral consumption) were separated by centrifugation for 10 min at a relative centrifugal force (RCF) of 2500 g. Concentrations of free Trp, tyrosine (TYR), phenylalanine (PHE), valine (VAL), leucine (LEU), and isoleucine (ILE) in plasma were analyzed by Biochrom 30 Plus amino acid analyzer (Biochrom Ltd., UK). VAL, LEU, and ILE levels were analyzed to calculate the ratio of plasma Trp to other large neutral amino acids (LNAAs). 5-HT depletion magnitude was calculated using the ratio of TRYP/ΣLNAAs before ATD at baseline minus T1 (after 4 h, immediately before fMRI experiment) (ATD baseline: TRYP/ΣLNAAs − ATDT1: TRYP/ΣLNAAs). The 5-HT supplementation magnitude was calculated using the ratio of TRYP/ΣLNAAs after TRYP supplementation at baseline minus T1 (TRYP + baseline: TRYP/ΣLNAAs − TRYP + T1: TRYP/ΣLNAAs).

### Pain testing

The pain testing block was composed of (i) pain threshold assessments (heat and pressure pain) followed by (ii) the assessment of facial and subjective responses to phasic heat stimulation (with intensities tailored to the individual pain threshold) and concluded (iii) with a questionnaire assessing situational pain catastrophizing.

#### Pain thresholds

##### Heat pain threshold

Thermal stimulation was applied to the left and right lateral lower leg by use of a Peltier-based, computerized thermal stimulator with a 3 × 3 cm^2^ contact probe (Medoc TSA-2001; Medoc Ltd, Ramat Yishai, Israel). Heat pain thresholds were determined using the method of limits. The temperature increased from 35 °C at a rate of 1 °C/s until the participants felt the stimulus to be barely painful and responded by pressing a stop button. Each time they pressed the button, the temperature returned to 35 °C and the next trial started after an inter stimulus interval (ISI) of 20–25 s. Following two familiarization trial, there were four test trials and the average of these trials was used to constitute the threshold estimate. This procedure was conducted on both legs, with the order of stimulation sides being randomized across participants.

##### Pressure pain threshold

Pressure stimuli were delivered by experienced investigators (MK, AJK) using a handheld pressure algometer (Algometer type II, Somadic Sales AB, Hörby, Sweden) with a probe area of 1 cm^2^. Stimulation sites where the left as well as the right volar forearm. Steady support was guaranteed by placing the participants’ arm on the table in front of the participant. Pressure pain thresholds were assessed using the method of limits. The pressure increased at a rate of 50 kPa/s until the participants felt the stimulus to be barely painful and responded by pressing a stop button (ISI 20–25 s). For each stimulation side, two practice trials were presented followed by four trials which were averaged to deliver estimates of pressure pain thresholds. Again, the order of stimulation sides was randomized across participants.

For further analyses, heat and pressure pain thresholds were averaged across right and left body sides, respectively.

#### Facial and subjective responses to non-painful and painful heat stimulation

##### Phasic heat stimulation

Ten non-painful (− 1 °C below the pain threshold, right body side) and 10 painful (+ 3 °C above the pain threshold, right body side) heat stimuli (Medoc TSA-2001; Medoc Ltd, Ramat Yishai, Israel) were applied on the right lateral lower leg in a pseudo-randomized order. Each phasic heat stimulus (painful/non-painful) had the same characteristics (5 s (plateau); rate of change: 4 °C/s; baseline temperature: 38 °C; ISI of 15–20 s).

##### Subjective responses

Following each stimulus, participants were asked by the experimenter to provide a verbal pain intensity rating using a numerical rating scale (NRS; 0–100). Participants were instructed that “0” corresponded to “no pain” and “100” to “extremely strong pain,” respectively.

##### Facial responses

The face of the participants was videotaped throughout the phasic heat stimulation procedures. The camera was located approximately 1.5 m from the participant. To mark the plateau phase of the stimuli, a LED visible to the camera, but not to the participant, was lit concurrently with the 5 s-thermal stimulation, starting when the target temperature was reached. During stimulation, participants were instructed not to talk and to look at a cross on the wall behind the camera to ensure that the face would always be recorded in an upright and frontal view. Facial expressions were coded from the video recordings using the Facial Action Coding System (FACS; Ekman and Friesen 1987), which is based on anatomical analysis of facial movements and distinguishes 44 different Action Units (AUs) produced by single muscles or combinations of muscles. Two certified FACS coder (qualified by passing an examination given by the developers of the system) who were blind to the condition (ADT vs. control drink) identified the frequency and the intensity (5-point scale) of the different Action Units. Inter-rater reliability using the Ekman formula (Ekman & Friesen 1987) was 0.82, which compares favorably to previous studies (Kunz et al. 2008a, b, 2012). A software designed for the analysis of observational data (the Observer Video-Pro; Noldus Information Technology) was used to segment the videos and to enter the FACS codes into a time-related database. Time segments of 7 s (5 s beginning just after the stimulus had reached the target temperature at plateau + 2 s for return to baseline) were selected for scoring. In total, 20 segments of thermal stimulation (10 non-painful and 10 painful segments) were analyzed for each participant.

For the purpose of necessary data reduction, we fused AU-frequency and AU-intensity values of each Action Unit by computing the product term (frequency × intensity) and also combined similar facial responses as has been done in preceding studies (Kunz et al. 2019). Those combinations include AU1_2, AU6_7, AU9_10, and AU25_26_27. For further analyses, we computed composite scores of “pain-relevant AUs” and of “other AUs”. This distinction was based on our previous review article on facial expressions of pain (Kunz et al. 2019). “Pain-relevant AUs” encompassed AU4, AU6_7, AU9_10, AU25_26_27, and “other AUs” encompassed all remaining AUs. Composite scores of “pain-relevant AUs” and “other AUs” were computed by averaging the respective AUs. Composite scores for “pain-relevant AUs” and “other AUs” were calculated separately for each condition (ATD, control) and separately for non-painful and painful heat stimulation.

### Affective state

In order to assess whether ATD might affect catastrophic thinking related to the experimental pain induction, we assessed a situational version of the Pain Catastrophizing Scale PCS (S‐PCS) (Campbell et al. 2010) after each session (ADT & control). Participants were instructed to reflect on thoughts or feelings during the past experience of the experimental pain stimuli. The scale contains 6 items that are rated on a five-point scale, with the end points “not at all” and “all the time.” In this study, the internal consistency of the S-PCS was good (control: *α* = 0.87; ADT: *α* = 0.79). In addition to the S-PCS, we used the Self-Assessment Manikin (SAM) to measure mood ratings (Lang 1980). The SAM is a pictorial affective rating system and ranges from a smiling, happy figure (− 4) to a sad figure (+ 4) when representing the valence dimension (Bradley and Lang 1994), indicative of mood.

### Statistical analysis

Analyses were conducted with SPSS 28 (IBM SPSS Statistics) and findings were considered to be statistically significant at *α* < 0.05. Shapiro–Wilk test was conducted to test for normality of distribution. In case of significant effects in the ANOVAs, Bonferroni-corrected post-hoc *t*-tests were calculated.

#### Manipulation check (analysis of plasma tryptophan)

Tryptophan concentration values were not normally distributed in the ATD testing session; thus, the Wilcoxon signed-rank test was used to assess changes in concentration levels in the ATD and control testing sessions.

#### Affective state

To investigate the effect of ATD on mood and situational pain catastrophizing, SAM mood ratings (4-h post-oral consumption of the ADT/control drink) and S-PCS scores (assessed at the end of the pain testing) were compared between ATD and control testing session using paired *t*-tests.

#### Pain thresholds

Paired *t*-tests were used to compare heat and pressure pain thresholds, respectively, between ATD and control condition.

#### Subjective responses

An ANOVA with repeated measures (within-subject factors: heat intensity (non-painful, painful) and condition (ATD, control)) was conducted to investigate the effect of ATD on pain intensity ratings.

#### Facial responses

ANOVAs with repeated measures (within-subject factors: heat intensity (non-painful, painful) and condition (ATD, control)) were conducted to investigate the effect of ATD on facial responses. Analyses were conducted separately for “pain relevant AUs” and for “other AUs.” Given that the facial response data were not normally distributed (left-skewed), the data was square-root transformed, as done in previous studies (Karmann et al. 2014; Kunz et al 2012).

## Results

### Plasma tryptophan (manipulation check)

In the ATD condition, tryptophan (Trp) concentration significantly decreased 4-h post-oral consumption (mean: 4.6 ± 3.9 µmol/l) compared to baseline levels (mean: 47.4 ± 11.6 µmol/l) (Wilcoxon signed-rank test *Z* =  − 4.5, *p* < 0.001). In the control condition, a significant increase (*Z* =  − 4.4, *p* < 0.001) in Trp concentration was detected (mean: 702.9 ± 442.9 µmol/l) compared to baseline (mean: 52.7 ± 13.8 µmol/l). The ratio of tryptophan to other large neutral amino-acids (LNAA’s), such as isoleucine, leucine, valine (Trp/LNAA)) is known to be a specific indicator of central 5-HT concentration. The Wilcoxon signed-rank revealed a significant decrease in the Trp/LNAA ratio in the ATD condition (*Z* =  − 4.5, *p* < 0.001) and a significant increase in this ratio in the control condition (*Z* =  − 4.4, *p* < 0.001) relative to the baseline level. The reduction in the Trp/LNAA ratio in the ATD condition relative to baseline was 92.8% (ATD: 0.008 ± 0.009 µmol/l; baseline: 0.12 ± 0.03 µmol/l). Thus, ATD resulted in a significant reduction in plasma tryptophan to levels known to markedly compromise central 5-HT neurotransmission.

### Affective state

**Fig. 1:**
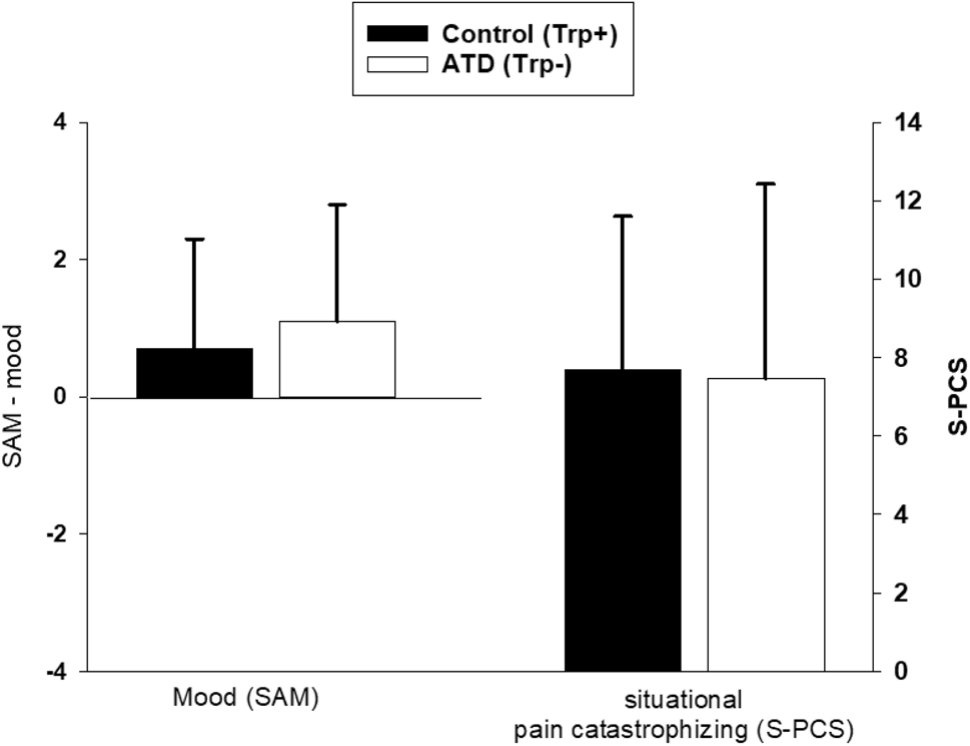
Effect of acute tryptophan depletion (ATD) on affective state indicators (mean, SD)

As can be seen in Fig. 1, ATD had no effect on mood (SAM valence scale; *p* = 0.836) nor on situational pain catastrophizing (S-PCS; *p* = 0.786) compared to the control condition.

### Pain thresholds

**Fig. 2:**
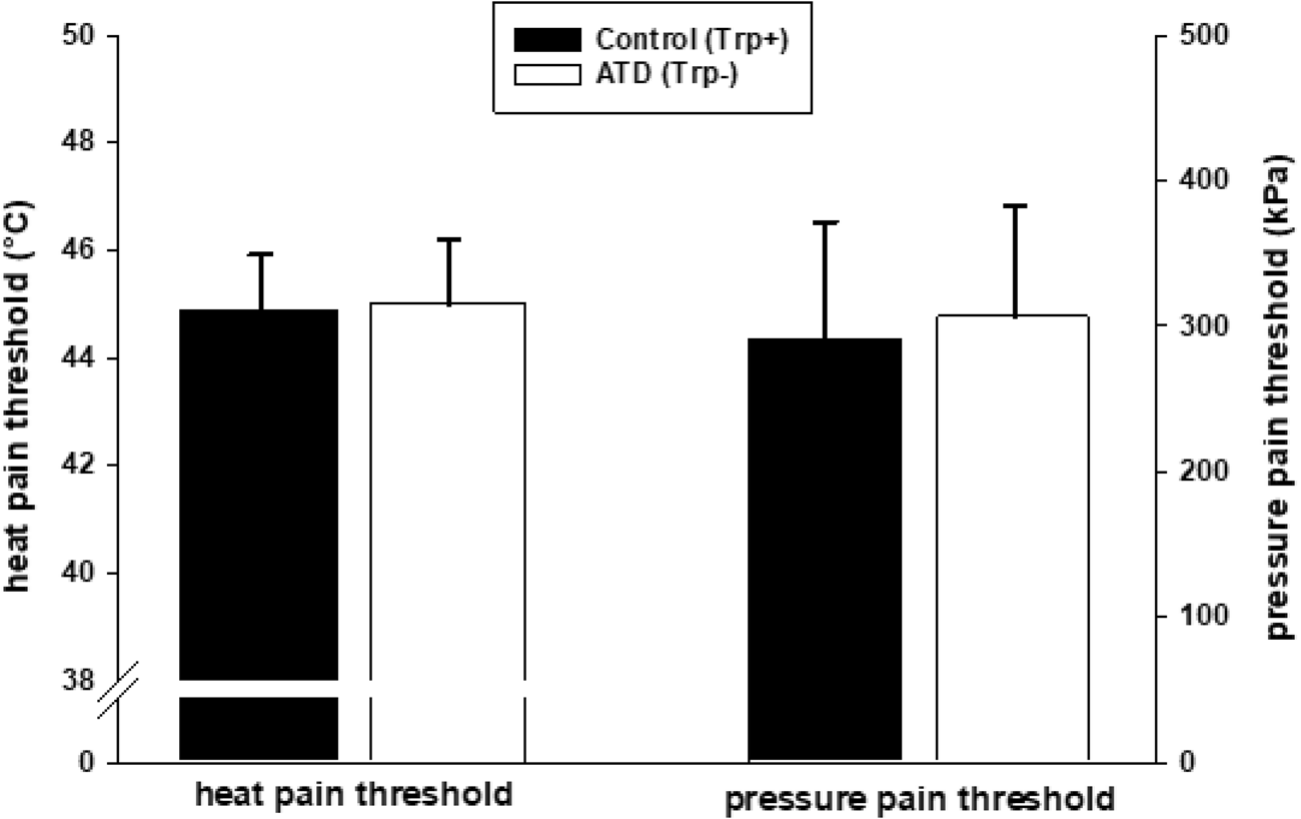
Effect of acute tryptophan depletion (ATD) on heat and pressure pain thresholds (mean, SD)

As can be seen in Fig. 2, neither heat pain thresholds (*T*(26) =  − 0.571, *p* = 0.573) nor pressure pain thresholds (*T*(26) = 0.122, *p* = 0.243) significantly differed between ATD and control condition.

### Subjective responses to phasic heat stimuli

**Fig. 3:**
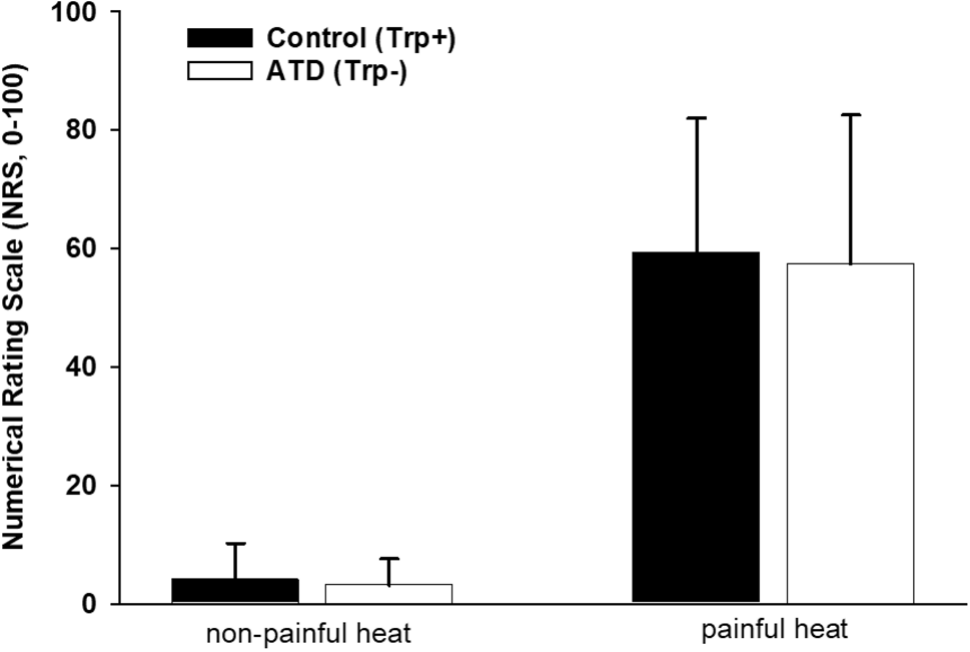
Effect of acute tryptophan depletion (ATD) on subjective responses to phasic heat stimuli of non-painful (− 1 °C below pain threshold) and painful (+ 3 °C above pain threshold) intensities (mean, SD)

As can be seen in Fig. 3, ATD had no effect on NRS ratings compared to the control condition (*F*(1,26) = 0.90; *p* = 0.351). Furthermore, there was no significant interaction between “condition” and “heat intensity” (*F*(1,26) = 0.16; *p* = 0.693). Only the factor “heat intensity” yielded a significant effect (*F*(1,26) = 176.01; *p* < 0.001), with intensities of + 3 °C above pain threshold being rated higher than the non-painful intensities (− 1 °C below pain threshold).

### Facial responses to phasic heat stimuli

The effect of ATD on facial responses was analyzed separately for pain-relevant AUs (AU4; AU6_7, AU9_10, AU25_26_27; Kunz et al. 2019) and for the remaining AUs (“other AUs”).

**Fig. 4:**
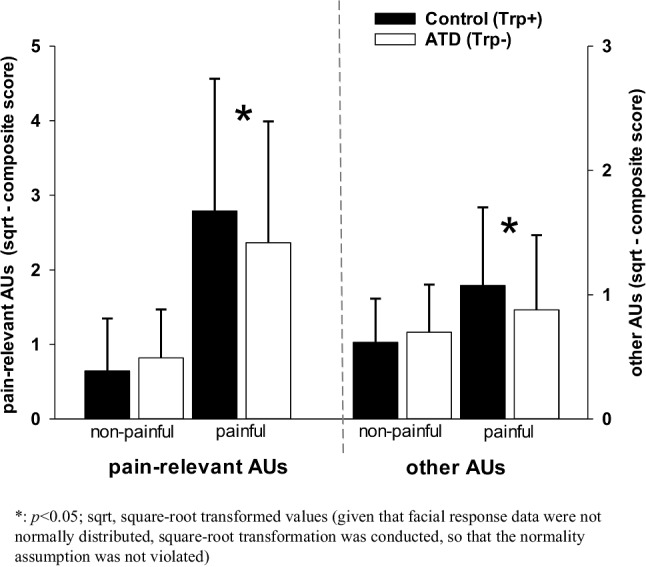
Effect of acute tryptophan depletion (ATD) on pain-relevant and other facial responses to phasic heat stimuli of non-painful (− 1 °C below pain threshold) and painful (+ 3 °C above pain threshold) intensities (mean, SD)

With regard to *pain-relevant AUs* (Fig. 4, left side), we found that pain-relevant facial responses significantly increased from non-painful to painful heat stimulation, given the main effect for “heat intensity” (*F*(1,26) = 46.35; *p* < 0.001). Although ATD had no overall significant effect on pain-relevant AUs (main effect for “condition” *F*(1,26) = 0.26, *p* = 0.612), we found a significant interaction between “condition” and “heat intensity” (*F*(1,26) = 12.24, *p* = 0.002). Bonferroni-corrected post-hoc *t*-tests were conducted separately for non-painful and painful heat stimuli. Whereas no effect of ATD was found for non-painful heat (*p* = 0.102), we found a significant decrease in pain-relevant facial responses to painful heat during ATD compared to the control condition (*p* = 0.040).

With regard to the *other (non-pain-relevant) AUs* (Fig. 4, right side), we found that the remaining AUs also significantly increased from non-painful to painful heat stimulation (*F*(1,26) = 10.64; *p* = 0.003). Although ATD had no overall significant effect on the other AUs (*F*(1,26) = 1.16, *p* = 0.292), we again found a significant interaction between “condition” and “heat intensity” (*F*(1,26) = 7.78, *p* = 0.010). Bonferroni-corrected post-hoc *t*-tests were conducted separately for non-painful and painful heat stimuli. Whereas no effect of ATD was found for non-painful heat (*p* = 0.262), we found a significant decrease in other facial responses to painful stimulation during ATD compared to the control condition (*p* = 0.014).

*In summary*, ATD led to a significant decrease in facial responses to painful stimulation that was apparent both for pain-relevant as well as for non-pain-relevant facial responses.

## Discussion

The present study examined whether the serotonergic system plays a role in facial expressions of pain by depleting plasma Trp concentrations in healthy volunteers via ATD in a double blind, placebo-controlled within-subject design. Blood analysis showed a substantial reduction in total plasma Trp levels and in the Trp/LNAA ratio after ATD; thus, indicating a markedly reduced central 5-HT neurotransmission (Crockett et al. 2012). We found no significant changes in pain thresholds, pain ratings, or in affective state indicators. Moreover, facial responses to pain were not—as hypothesized—increased but decreased after ATD compared to the control condition. We will discuss these findings in detail below.

### ATD effects on pain thresholds, pain ratings, and affective state

With regard to pain thresholds, we investigated ATD effects on two types of pain thresholds, namely heat as well as pressure pain thresholds. Using two different types of experimental pain modalities allows for assessing how reliable 5-HT effects are across two different pain modalities that partially target different nociceptor populations. Whereas heat stimuli mainly activate skin nociception, pressure stimuli target both skin and to a greater degree deep tissue nociceptors (Kosek et al. 1999; Yu and Mense 1990). Given that deeper tissue nociceptors might be more affected by descending serotonergic pathway, even greater ATD effects might be expected with regard to pressure pain thresholds. In contrast to our expectation, ATD neither affected heat nor pressure pain thresholds. To the best of our knowledge, the effect of ATD on pain thresholds has been investigated in three previous studies (Abbott et al. 1992; Martin et al. 2017, Trotter et al. 2022). In two of these studies, cold pressure pain was used to assess ATD related changes in pain thresholds and in both studies no significant changes in pain thresholds were found (Abbott et al. 1992; Trotter et al. 2022) which is an accordance with our findings. Only Martin and colleagues (2017) who similarly to us, used a heat pain protocol to assess pain thresholds, found a significant reduction of heat pain thresholds after ATD. More precisely, Martin used the staircase method to assess pain thresholds and found that pain thresholds decreased from a mean of 44.0 °C (control) to 43.03 °C (ATD) (Martin et al. 2017). In our study, we used the method of adjustment and found that heat pain thresholds remained stable with a mean of 44.9 °C for both ATD and control conditions. It is possible that methodological differences (e.g., staircase method vs. method of adjustment) or sample characteristics (e.g., different gender distribution) could explain the different findings. However, given that—including the present study—three out of four studies do not find ATD related changes in cold (Abbott et al. 1992; Trotter et al. 2022) and in heat and pressure pain thresholds (our study) is an indication that central reduction of 5-HT via ATD does not have a strong effect on pain threshold estimates.

Besides pain thresholds which target more the lower range of pain sensitivity, we also assessed pain intensity ratings of supra-threshold heat stimuli. We found that ATD also had no effect on pain intensity ratings of these stimuli. The supra-threshold intensities were tailored to the individual pain threshold (+ 3 °C) and were on average rated as eliciting moderate pain sensations. Considering supra-threshold pain intensities, previous ATD studies found reduced tolerance thresholds after ATD in two cases (Martin et al. 2017; Trotter et al. 2022). Thus, it is possible that central reduction of 5-HT mostly affects the upper range of pain sensitivity (tolerance thresholds), whereas the lower range or the midrange is less affected.

We also assessed the effect of ATD on mood and situational pain catastrophizing as indicators of potential changes in affective states. Again, no effect of ATD was found which is in accordance with previous findings, demonstrating that the effect of ATD on affective states seems negligible in healthy individuals with no history of depression (Young 2013).

### ATD effects on facial expressions of pain

We hypothesized that facial responses to painful stimuli are increased after ATD, given previous findings that ATD can lead to heightened pain sensitivity (Martin et al. 2017; Nagata et al. 2007) as well as to a disinhibition of (goal-oriented) behavioral responses (Walderhaug et al. 2002; 2008; Crockett et al. 2009). However, ATD did not result in an increase but in a decrease of facial responses to painful stimuli. This decrease in facial responses was not specific to pain-related facial responses but was also apparent for facial responses with little association to painful experiences. This separation of facial responses into pain-related and not-pain related is based on previous evidence showing that there is a subset of facial responses that is predominantly displayed during experimental and clinical pain states (Kunz et al. 2019; Prkachin 1992). Finding that lower levels of 5-HT led to a decrease in pain-relevant as well as in all other facial responses is directly contrary to our hypothesis but interestingly, a similar association between serotonin and facial expressiveness has recently been reported (Bershad et al. 2019). Bershad and colleagues found that the administration of N-Methyl-3, 4-methylenedioxyamphetamine (MDMA), which induces rapid 5-HT release, was linked to a nonspecific increase in zygomatic activation both to pleasant touch (slow stroking) as well as to less-pleasant touch (fast stroking) (Bershad et al. 2019). How can this positive association between 5-HT activity and affect-unspecific facial expressiveness be explained?

One possible explanation might be found in the effect of serotonin on social behavior. In human and rodent studies, low 5-HT levels have been shown to be linked to decreased social play, to dampened perception of social cues and to reduced social behavior (Kiser et al. 2012). Facial expressions of pain are social signals that have the potential to serve as a “social lubricate” by eliciting empathetic responses and help in others (Hadjistavropolous et al. 2011). It is thus, possible that the low 5-HT levels due to ATD in our study reduced the disposition for social behavior, including the disposition to facially communicate one’s pain experience to the social environment.

Another possible explanation might be the linkage between 5-HT and motoneuron activity in the brainstem. There is a widespread network of brain areas involved in generating facial responses to pain, including motor areas (M1, striatum), pain-related areas (S1, insula, anterior cingulate cortex), and areas involved in regulation of prosocial behavior (prefrontal areas) (Kunz et al. 2011). All the activity within these areas converges at the end on the motor nucleus of the facial nerve (cranial nerve VII) situated in the brainstem (Cattaneo & Pavesi 2014) to elicit facial responses. The excitability of motoneurons, including the motor nucleus of the facial nerve has been shown to be linked to 5-HT activity in animal studies (McCall & Aghajanian 1979; White & Neuman 1980). Interestingly, 5-HT does to directly lead to increased excitability but brings about this outcome indirectly by enhancing the effects of excitatory afferent inputs to the facial nucleus. McCall & Aghajanian (1979) could show that intravenous administration of 5-HT agonists or 5-HT releasing agents facilitated the effects of excitatory inputs (e.g., motor cortex stimulation) to facial motoneurons in rats. Thus, in our study the reduced central 5-HT might have led to a decreased facilitation of excitatory inputs to the facial motor neurons which resulted in reduced facial responses to pain.

### Limitations

There are some limitations to our study. First of all, the sample studied was of limited size and only included female participants, given that elevated behavioral as well as biochemical ATD responses have been found in females compared to males (Ellenbogen et al. 1996; Nishizawa et al. 1997). In case of more positive findings, verification for men and in a larger sample might become necessary that would also allow for more complex statistical analyses. Moreover, we analyzed facial responses using FACS which relies on visible facial movements and has the advantage that it allows to reliably differentiate between different facial muscles and muscle groups, respectively. However, it has the disadvantage that it is not able to capture subtle facial muscle activity that would have been better captured using electromyography (EMG). Therefore, the assessment of EMG might be promising in future studies to further investigate how ATD affects facial expressiveness.

## Conclusions

Using ATD to reduce central 5-HT levels in healthy females, we found a significant decrease in facial responses to experimental pain stimuli. Given that this decrease was not specific for pain-related facial responses and given that we found no ATD related changes in any of the other pain outcomes (pain thresholds as well as supra-threshold pain ratings) suggests that 5-HT mostly affects the last step of facial encoding of pain; possibly due to (i) a diminished disposition to display social behavior or due to (ii) a decreased facilitation of excitatory inputs to the facial motor neuron.

## References

[CR1] Abbott FV, Etienne P, Franklin KBJ, Morgan MJ, Sewitch MJ, Young SN (1992). Acute tryptophan depletion blocks morphine analgesia in the cold-pressor test in humans. Psychopharm.

[CR2] Aquili L (2020). The role of tryptophan and tyrosine in executive function and reward processing. Int J of Tryptoph Res.

[CR3] Bär KJ, Köhler S, de la Cruz F, Schumann A, Zepf FD, Wagner G (2020). Functional consequences of acute tryptophan depletion on raphe nuclei connectivity and network organization in healthy women. NeuroIm.

[CR4] Basbaum A, Fields H (1984). Endogenous pain control systems: brainstem spinal pathways and endorphin circuitry. Neurosci.

[CR5] Bershad AK, Mayo LM, Van Hedger K, McGlone F, Walker SC, de Wit H (2019). Effects of MDMA on attention to positive social cues and pleasantness of affective touch. Neuropsychopharmacol.

[CR6] Bradley MM, Lang PJ (1994). Measuring emotion: the self-assessment manikin and the semantic differential. J Behav Ther Exp Psychiatry.

[CR7] Campbell CM, Kronfli T, Buenaver LF, Smith MT, Berna C, Haythornthwaite JA, Edwards RR (2010) Situational versus dispositional measurement of catastrophizing: associations with pain responses in multiple samples. The Journal of Pain 11(5):443–453. 10.1016/j.jpain.2009.08.009 PMC2898132 20439057

[CR8] Carpenter LL, Anderson GM, Pelton GH, Gudin JA, Kirwin PD, Price LH, McDougle CJ (1998). Tryptophan depletion during continuous CSF sampling in healthy human subjects. Neuropsychopharmacol.

[CR9] Cattaneo L, Pavesi G (2014). The facial motor system. Neuroscience Biobehav Rev.

[CR10] Cools R, Roberts AC, Robbins TW (2008). Serotoninergic regulation of emotional and behavioural control processes. Trends Cogn Sci.

[CR11] Cooper TE, Heathcote LC, Clinch J, Gold JI, Howard R, Lord SM, Schechter N, Wood C, Wiffen PJ (2017) Antidepressants for chronic non‐cancer pain in children and adolescents. Cochrane Database of Systematic Reviews 8 10.1002/14651858.CD012536.pub2 PMC6424379 28779491

[CR12] Craig KD, Prkachin KM, Grunau RVE, Turk DC, Melzack R (2011). The facial expression of pain. Handbook of pain assessment.

[CR13] Crockett MJ, Clark L, Robbins TW (2009). Reconciling the role of serotonin in behavioral inhibition and aversion: acute tryptophan depletion abolishes punishment-induced inhibition in humans. J Neurosci.

[CR14] Crockett MJ, Clark L, Roiser JP, Robinson OJ, Cools R, Chase HW, Robbins TW (2012). Converging evidence for central 5-HT effects in acute tryptophan depletion. Mol Psychiatry.

[CR15] Ekman P, Friesen WV (1987). Facial action coding system.

[CR16] Ellenbogen MA, Young SN, Dean P, Palmour RM, Benkelfat C (1996). Mood response to acute tryptophan depletion in healthy volunteers: sex differences and temporal stability. Neuropsychopharmacol.

[CR17] Evers EAT, Tillie DE, van der Veen FM, Lieben CK, Jolles J, Deutz NEP (2005). Effects of a novel method of acute tryptophan depletion on plasma tryptophan and cognitive performance in healthy volunteers. Psychopharmacol.

[CR18] Feng W, Ming GU, Yu-Xia C (2012). New tricks for an old slug: descending serotonergic system in pain. Acta Physiologica Sinica.

[CR19] Goesling J, Clauw DJ, Hassett AL (2013). Pain and depression: an integrative review of neurobiological and psychological factors. Curr Psychiatry Rep.

[CR20] Hadjistavropoulos T, Craig KD, Duck S, Cano A, Goubert L, Jackson PL, Fitzgerald TD (2011). A biopsychosocial formulation of pain communication. Psychol Bulletin.

[CR21] Karmann AJ, Lautenbacher S, Bauer F, Kunz M (2014) The influence of communicative relations on facial responses to pain: does it matter who is watching? Pain Research and Management 19:15–22 10.1155/2014/195286 PMC3938338 24432350

[CR22] Karmann AJ, Lautenbacher S, Kunz M (2015). The role of inhibitory mechanisms in the regulation of facial expressiveness during pain. Biol Psychol.

[CR23] Karmann AJ, Maihöfner C, Lautenbacher S, Sperling W, Kornhuber J, Kunz M (2016). The role of prefrontal inhibition in regulating facial expressions of pain: a repetitive transcranial magnetic stimulation study. J Pain.

[CR24] Kiser D, SteemerS B, Branchi I, Homberg JR (2012). The reciprocal interaction between serotonin and social behaviour. Neurosci Biobehav Rev.

[CR25] Kosek E, Ekholm J, Hansson P (1999). Pressure pain thresholds in different tissues in one body region. The influence of skin sensitivity in pressure algometry. Scand J Rehab Med.

[CR26] Kunz M, Scharmann S, Hemmeter U, Schepelmann K, Lautenbacher S (2007). The facial expression of pain in patients with dementia. Pain.

[CR27] Kunz M, Chatelle C, Lautenbacher S, Rainville P (2008). The relation between catastrophizing and facial responsiveness to pain. Pain.

[CR28] Kunz M, Mylius V, Schepelmann K, Lautenbacher S (2008). Impact of age on the facial expression of pain. J Psychosom Res.

[CR29] Kunz M, Chen J-I, Lautenbacher S, Vachon-Presseau E, Rainville P (2011). Cerebral regulation of facial expressions of pain. J Neuroscience.

[CR30] Kunz M, Faltermeier N, Lautenbacher S (2012). Impact of visual learning on facial expressions of physical distress: a study on voluntary and evoked expressions of pain in congenitally blind and sighted individuals. Biol Psychol.

[CR31] Kunz M, Meixner D, Lautenbacher S (2019). Facial muscle movements encoding pain—a systematic review. Pain.

[CR32] Kunz M, Chen J-I, Rainville P (2020). Keeping an eye on pain expression in primary somatosensory cortex. Neuroimage.

[CR33] Lang P (1980) Behavioral treatment and bio-behavioral assessment: computer applications. Technology in mental health care delivery systems, 119–137

[CR34] Larochette AC, Chambers CT, Craig KD (2006). Genuine, suppressed and faked facial expressions of pain in children. Pain.

[CR35] Martin SL, Power A, Boyle Y, Anderson IM, Silverdale MA, Jones AK (2017). 5-HT modulation of pain perception in humans. Psychopharmacol.

[CR36] Martins D (2019) Serotonin and nociception: from nociceptive transduction at the periphery to pain modulation from the brain. In The Serotonin System (pp. 203–224). Academic Press

[CR37] McCall RB, Aghajanian GK (1979). Serotonergic facilitation of facial motoneuron excitation. Brain Res.

[CR38] Nagata E, Hamada J, Shimizu T, Shibata M, Suzuki S, Osada T, Takaoka R, Kuwana M, Suzuki N (2007). Altered levels of serotonin in lymphoblasts derived from migraine patients. Neurosc Res.

[CR39] Nishizawa S, Benkelfat C, Young SN, Leyton M, Mzengeza SD, De Montigny C, Diksic M (1997). Differences between males and females in rates of serotonin synthesis in human brain. PNAS.

[CR40] Prkachin KM (1992). The consistency of facial expressions of pain: a comparison across modalities. Pain.

[CR41] Sheehan DV, Lecrubier Y, Sheehan KH, Amorim P, Janavs J, Weiller E, Dunbar GC (1998). The Mini-International Neuropsychiatric Interview (MINI): the development and validation of a structured diagnostic psychiatric interview for DSM-IV and ICD-10. J Clin Psychiatry.

[CR42] Trotter PD, Smith SA, Moore DJ, O’Sullivan N, McFarquhar MM, McGlone FP, Walker SC (2022). Acute tryptophan depletion alters affective touch perception. Psychopharmacol.

[CR43] Walderhaug E, Lunde H, Nordvik JE, Landrø N, Refsum H, Magnusson A (2002). Lowering of serotonin by rapid tryptophan depletion increases impulsiveness in normal individuals. Psychopharmacol.

[CR44] Walderhaug E, Landrø NI, Magnusson A (2008). A synergic effect between lowered serotonin and novel situations on impulsivity measured by CPT. J Clin Exp Neuropsychol.

[CR45] White SR, Neuman RS (1980). Facilitation of spinal motoneurone excitability by 5-hydroxytryptamine and noradrenaline. Brain Res.

[CR46] Williams WA, Shoaf SE, Hommer D, Rawlings R, Linnoila M (1999). Effects of acute tryptophan depletion on plasma and cerebrospinal fluid tryptophan and 5-hydroxyindoleacetic acid in normal volunteers. J Neurochemistry.

[CR47] Young SN (2013). Acute tryptophan depletion in humans: a review of theoretical, practical and ethical aspects. J Psychiatry Neurosci.

[CR48] Young SN, Smith SE, Pihl RO, Ervin FR (1985). Tryptophan depletion causes a rapid lowering of mood in normal males. Psychopharmacol.

[CR49] Yu XM, Mense S (1990). Response properties and descending control of rat dorsal horn neurons with deep receptive fields. Neurosci.

